# Advancing bioinformatics capacity through Nextflow and nf-core: lessons from an early-to mid-career researchers–focused program at The Kids Research Institute Australia

**DOI:** 10.3389/fbinf.2025.1610015

**Published:** 2025-08-29

**Authors:** Patricia Agudelo-Romero, Talya Conradie, Jose Antonio Caparros-Martin, David Jimmy Martino, Anthony Kicic, Stephen Michael Stick, Christopher Hakkaart, Abhinav Sharma, the Theme Collaboration Group

**Affiliations:** ^1^ Wal-Yan Respiratory Research Centre, The Kids Research Institute Australia, Perth, WA, Australia; ^2^ Australian Research Council Centre of Excellence in Plant Energy Biology, School of Molecular Sciences, The University of Western Australia, Perth, WA, Australia; ^3^ European Virus Bioinformatics Center, Friedrich-Schiller-Universitat Jena, Jena, Thuringia, Germany; ^4^ Curtin Health Innovation Research Institute (CHIRI), Curtin University, Perth, WA, Australia; ^5^ School of Medicine, The University of Western Australia, Perth, WA, Australia; ^6^ School of Population Health, Curtin University, Perth, WA, Australia; ^7^ Centre for Cell Therapy and Regenerative Medicine, Medical School, The University of Western Australia, Perth, WA, Australia; ^8^ Department of Respiratory and Sleep Medicine, Perth Children’s Hospital, Perth, WA, Australia; ^9^ Seqera Labs, Barcelona, Spain; ^10^ DSI-NRF Centre of Excellence for Biomedical Tuberculosis Research, SAMRC Centre for Tuberculosis Research, Division of Molecular Biology and Human Genetics, Faculty of Medicine and Health Sciences, Stellenbosch University, Cape Town, Western Cape, South Africa

**Keywords:** bioinformatics, capacity building, nextflow, early-mid career researcher, omics, pipelines, nf-core

## Abstract

The increasing adoption of high-throughput “omics” technologies has heightened the demand for standardized, scalable, and reproducible bioinformatics workflows. Nextflow and nf-core provide a robust framework for researchers, particularly early- and mid-career researchers (EMCRs), to navigate complex data analysis. At The Kids Research Institute Australia, we implemented a structured approach to bioinformatics capacity building using these tools. This perspective presents nine practical rules derived from lessons learnt, which facilitated the successful adoption of Nextflow and nf-core, addressing implementation challenges, knowledge gaps, resource allocation, and community support. Our experience serves as a guide for institutions aiming to establish sustainable bioinformatics capabilities and empower EMCRs.

## Introduction

The demand for researchers skilled in bioinformatics has surged due to the increasing adoption of high-throughput “omics” technologies, presenting both opportunities and challenges. Researchers without formal training in programming or workflow engines must now analyze high-dimensional datasets using various tools, while ensuring analyses are reproducible across computational environments. This has highlighted a need for omics data workflows that are standardized, scalable, and reproducible. In this context, we aimed to provide early- and mid-career researchers (EMCRs) with opportunities to gain knowledge and establish a bioinformatics support system (Woelmer et al., 2021) using workflow managers like Nextflow and the nf-core community.

Nextflow is a workflow management system designed to streamline bioinformatics research by composing multiple tools into a single pipeline (Di Tommaso et al., 2017). Its flexibility, portability and scalability ranges from local servers, high-performance clusters, and cloud environments with minimal reconfiguration. Nextflows native task parallelization, makes it ideal for handling high-volume of datasets originating from modern “omics” technologies (Dai and Shen, 2022). In addition, the nf-core community enhances the Nextflow ecosystem by connecting users through hackathons, seminars, training, and collaborative platforms like Slack (Ewels et al., 2020; Langer et al., 2024). For newcomers, this support is invaluable for troubleshooting and professional growth. Additionally, the nf-core community develops and maintains pipelines used across different fields of life sciences. Each pipeline follows strict guidelines to ensure robustness, reproducibility, and ease of use, which are key features for EMCRs entering bioinformatics.

For institutions and researchers in “omics” studies, adopting Nextflow and nf-core is strategic. These tools enable management of high-throughput data, effective collaboration, and reproducible results, ensuring continued scientific advancement. This is important for EMCRs driving innovation. By integrating these tools into training, institutions can better prepare researchers to meet modern bioinformatics challenges (Stephens et al., 2015).

**FIGURE 1:**
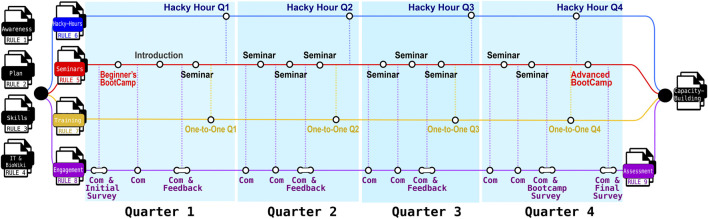
Overview of the nine practical rules derived from lessons learnt in building sustainable bioinformatics capacity using Nextflow and nf-core, with a focus on early- to mid-career researchers (EMCRs). Based on our experience at The Kids, the process begins with four foundational steps: Rule 1 (Develop a Strategic Plan), Rule 2 (Promote Institutional Awareness), Rule 3 (Identify Needs and Skill Gaps), and Rule 4 (Engage with IT and Documentation). Subsequent implementation activities are reflected across the timeline and aligned with: Rule 5 (Regular seminars), Rule 6 (Hacky-Hours), and Rule 7 (Structured and Personalized Training), which mark key milestones achieved each quarter. The initiative is further supported by ongoing efforts to strengthen participation and sustainability through Rule 8 (Facilitate Continuous Learning and Engagement) and Rule 9 (Assess Program Design and Implementation). Communication (Com). Program materials and resources are publicly available via the Nextflow-BioWiki (https://github.com/TelethonKids/Nextflow-BioWiki) (Agudelo-Romero et al., 2023).

Here, we share our experience at The Kids Research Institute Australia (The Kids), a research institution in Perth (Australia) with a diverse workforce and a strong commitment to building EMCR expertise in computational biology. The Theme Collaboration Group, a multidisciplinary team across The Kids, played a key role in shaping this initiative (Supplemental Data S1). We outline essential, practical rules based on lessons learnt, that supported the successful adoption and implementation of Nextflow (Table 1). These guidelines aim to help other institutions establish sustainable and robust bioinformatics capabilities, enabling researchers to fully leverage Nextflow and nf-core for scientific discovery. Figure 1 summarizes the key rules and the timeline of routine events critical to Nextflow adoption.

**TABLE 1:** Summary of the motivation for the rules’ development.

Motivation	Rule
Implementation	Rule 1 and 2
Knowledge gap	Rule 3
Resources	Rule 4
Support	Rule 5, 6, 7 and 8
Engagement and evaluation	Rule 9

## Rule 1: Develop a strategic plan aligned to the institutional priorities

A well-designed capacity development program within any institute should (i) reflect the needs of the institution and (ii) adhere to a well-scoped curriculum that benefits the entire organization. Identifying core stakeholders and aligning the program with the institution’s current and future data analysis needs is essential for ensuring its long-term relevance and impact (Aron et al., 2021).

At The Kids, our vision is “Happy Healthy KIDS,” and our objective is to enhance the health, development, and lives of children and adolescents through exemplary research. Our work encompasses a wide array of disciplines, from fundamental to applied science. In light of this diversity, we have concentrated on research streams dependent on omics data processing, providing training for students and EMCRs engaged in these data-intensive fields. We hosted an internal town hall meeting, attended by research teams engaged in bioinformatics, as well as members of the Pawsey Supercomputing Research Centre, an Australian government-supported high-performance computing (HPC) facility. During this meeting, we documented common roadblocks and challenges experienced by bioinformatic users, which laid the foundation for developing a strategic plan and the formation of a special interest group (SIG) with focus on bioinformatics. Building on these insights, we established a three-pillar strategy aimed at improving the efficiency, repeatability, and scalability of our bioinformatics investigations. This strategy focuses on transitioning from *ad hoc* shell scripts to the integration of Nextflow workflow manager into our computational research (Sztuka et al., 2024) while making the best use of shared resources.

1. Pillar one of our strategy involved identifying available computational resources, understanding their allocation across the different research groups, and assessing existing knowledge gaps and bioinformatics needs.
2. Pillar two focused on promoting a community of “power users” by integrating support and mentorship. A mentorship program was established to guide EMCRs through their learning journey, while promoting collaboration, a cornerstone of data-driven research. Researchers were encouraged to share their experiences, common challenges, and best practices, ultimately strengthening knowledge exchange, and sparking innovation with a strong, supportive community.
3. Pillar three emphasized adherence to established standards and best practices. We promoted the use of bioinformatics standards and provided templates and guidelines for creating reproducible and well-documented Nextflow pipelines, thereby enhancing research quality and credibility.

These three pillars informed the design of engagement activities, including seminars and online tutorials (Rule 5), Hacky-Hours (Rule 6), and one-one-one sessions and bootcamps (Rule 7). Additionally, continuous evaluation and feedback (Rule 8 and 9) were pivotal in refining and adapting these activities to meet the evolving needs of researchers effectively.

## Rule 2: Promote institutional awareness of reproducible, robust and open science

Promoting institutional awareness of reproducible data analysis and bioinformatics best practices is crucial in “omics” fields like transcriptomics, genomics, proteomics, metabolomics, and microbiomics. As researchers increasingly adopt “multi-omics” approaches to gain deeper insights into biological phenomena (Reel et al., 2021; Vahabi and Michailidis, 2022), the importance of reproducibility, robustness and open science cannot be overstated. These practices ensure reliable and actionable research findings while promoting transparency and allowing for validation, which is vital for scientific progress (Kraus, 2014; Stewart et al., 2022). A successful implementation of the capacity-building strategy (Rule 1) should ensure clear communication of the best bioinformatic practices. Participants must understand the importance of these practices in programming and computational experiments, such as testing simulations, algorithms, and models. Additionally, they should recognize the clear benefits of applying these practices within the context of their own work and research groups. (Carey and Papin, 2018; Heise et al., 2023).

At The Kids, we accomplished this through (i) direct approaches, using seminars and Hacky-Hours, as well as (ii) indirectly through mailing lists and a dedicated Microsoft Teams channel. These institutional platforms allowed us to share relevant resources and promote awareness effectively to embed reproducibility and open science practices.

## Rule 3: Identify the needs and skill gaps of different research groups within the institute

The key to successful capacity building effort lies in understanding existing skills and identifying future needs of research groups (Woelmer et al., 2021). We began by conducting an initial survey aimed at comprehensively evaluating key requirements of the EMCRs (Supplemental Data S2). The survey gathered information on (i) basic computational skills (e.g., Linux command line proficiency or scripting using Python and/or R languages), (ii) familiarity with bioinformatics tools and workflows; (iii) experience with Nextflow and nf-core; (iv) and specific challenges faced in their research programs.

We encouraged participation by emphasizing the importance of the surveys in identifying bioinformatic pipeline needs at The Kids, with weekly follow-ups over 4 weeks. This approach ensured detailed and honest responses. The results provided an accurate overview of the current bioinformatics capabilities and needs, and these insights shaped our implementation strategy, helping us identify priority topics for training and skill development.

Although the surveys were conducted as internal consultation and not designed for formal data analysis, it provided valuable insight that guided the development of the training. Using insights from the survey, we categorized researchers’ skills into three levels (beginner, intermediate, and advanced). This allowed us to design targeted training programs to address the identified gaps by building upon the sensitize, train, hack and collaborate model (Karega et al., 2023).

1. Beginner users focused on basic skills and attended introductory courses on Linux, bioinformatics fundamentals (Brandies and Hogg, 2021), and monitoring pipelines using the Seqera Platform (https://seqera.io/platform/).
2. Intermediate users focused on workflow development with Nextflow and nf-core pipeline templates (Roach et al., 2022).
3. Advanced users specialized in customizing existing nf-core pipelines and integrating new tools for cluster systems, such as those provided within Australia by Australian Pawsey Supercomputing Research Centre (Pawsey Supercomputing Research Centre PerthW. Australia, 2023a; Pawsey Supercomputing Research Centre PerthW. Australia, 2023b; Pawsey Supercomputing Research Centre PerthW. Australia, 2023c) and the Australian BioCommons Leadership Share (ABLeS) program (Gustafsson et al., 2023).

This targeted approach ensured that researchers received the precise support they needed to advance their bioinformatics skills and progress efficiently within their research projects.

## Rule 4: Engage with the IT team for infrastructure automation and sustainability through documentation (Nextflow-Biowiki)

Building a sustainable bioinformatics environment requires close collaboration with the Information Technology (IT) team. Their expertise in infrastructure management, data storage (short and long-term), and data sharing and protection is invaluable for adhering to responsible big data research practices (Zook et al., 2017). At The Kids, we partnered with the IT team to standardize and streamline frequent computational requests for “omics” data analysis and to establish long-term storage solutions for the Nextflow-Biowiki documentation created during the program (Agudelo-Romero et al., 2023).

To optimize infrastructure, the IT team developed a baseline virtual machine (VM) template. This template standardized configurations for efficient installation of the core dependencies, including Java (LTS version) (Arnold et al., 2005), Nextflow (Di Tommaso et al., 2017), and nf-core (Ewels et al., 2020). Package managers like Bioconda (Dale et al., 2018) and Mamba (mamba-Org, n. d.), and containerization tools such as Docker (da Veiga Leprevost et al., 2017) and Singularity (Kurtzer et al., 2017), were also preconfigured. Shared access to critical resources (e.g., genome reference files) was enabled, and computing resources, such as number of CPU cores, memory and disk space (scratch workspaces), were allocated based on project-specific data and analysis needs. To ensure robustness, we validated the setup by running various nf-core pipelines, addressing any issues and confirming consistent performance. We also ensured sufficient storage for output files generated on the VMs. At our institution, each user is provided with 3,000 GB of scratch spaces, however, actual storage requirements vary depending on several factors, including the number and size of the input and output files, as well as whether intermediate files are retained during pipeline execution.

At the same time, to ensure long-term sustainability, we created comprehensive documentation that could be used for future reference for EMCR and researchers’ community. The Nextflow-BioWiki project (Agudelo-Romero et al., 2023) is hosted on The Kids’ institutional GitHub repository (https://github.com/TelethonKids), where it remains publicly accessible and actively maintained. This resource includes detailed guides on bioinformatics tools, workflows execution, troubleshooting in our infrastructure, online manuals, video tutorials, and Frequently Asked Questions (FAQs); catering to diverse learning styles. The documentation was designed to be user-friendly and accessible to all researchers. Regular updates based on users’ feedback have ensured that resources remain relevant and aligned with technological advancements and community needs.

## Rule 5: Conduct regular seminars using relevant nf-core pipelines

Regular seminars aimed to introduce participants to conducting computational experiments under optimal conditions (“the happy path”), focusing on data analysis and interpretation. For the implementation of monthly seminars, we followed principles outlined by Fadlelmola et al., 2019. Seminars were designed to address real-world data analysis challenges, such as parameter optimization and chaining multiple pipelines for comprehensive analyses.

At The Kids, regular seminars covered the breadth of available resources, as key references, without overwhelming participants. These regular seminars also offered a unique opportunity to increase awareness about the resources available from the Institute and the Nextflow and nf-core communities, such as pipeline-specific “byte size” talks. To facilitate focused learning, we organized dedicated seminars for “omics” pipelines under cohesive themes, including:

1. Transcriptomics: nf-core/rnaseq (Patel et al., 2024), nf-core/differentialabundance (Wacker et al., 2023), nf-core/smrnaseq (Peltzer et al., 2024b).
2. Methylation: nf-core/methylseq (Ewels et al., 2024), nf-core/atacseq (Patel et al., 2023).
3. Microbiome: nf-core/ampliseq (Straub et al., 2024), nf-core/mag (Yates et al., 2024), agudeloromero/everest_nf (Agudelo-Romero et al., 2025).
4. Single-cell: nf-core/scrnaseq (Peltzer et al., 2024a).

This thematic grouping allowed participants to dive deeper into specific areas, establishing a better understanding of key biological concepts related to the “omics” strategies, while also encouraging collaborations among students and EMCRs working on similar topics.

Ultimately, during our seminars, we encouraged participants to explore and embrace cloud platforms as an integral part of their continued growth. Offering auxiliary opportunities for talent enhancement and performing their data analysis in the cloud. Consequently, certain participants in this program obtained research credits for performing their analysis and deploying Nextflow on cloud infrastructure. Supplementary Table S1 delineates cloud computing companies that extend credits for academic purposes.

## Rule 6: Organize practical training sessions (Hacky-Hours)

While regular seminars introduce theoretical concepts, Hacky-Hours translate them into practical skills through coordinated sessions tailored to the specific needs of EMCRs (Heise et al., 2023). These sessions provide hands-on opportunities to address challenges such as accessing different infrastructures or optimizing computing sources for diverse projects, empowering EMCRs to build their analytical capacity effectively.

The involvement of the IT team and researchers in designing Hacky-Hours provided valuable insight in the sessions. The IT team contributed by offering guidance on structured use of resources, ensuring efficient resource allocation. Meanwhile, engaging with researchers helped identify opportunities to refine the program’s content. For instance, when a specific nf-core pipeline was routinely used, an interactive Hacky-Hour session was organized on that pipeline. This session included step-by-step instructions and troubleshooting, delivering practical and targeted training (Boudabous and Tekaia, 2020).

At The Kids, bi-weekly Hacky-Hours (30–90 min) were structured around topics identified via participant surveys. Initially, these sessions focused on fundamental skills such as downloading and configuring nf-core pipelines. As Nextflow and nf-core adoption grew, surveys revealed a growing need for advanced topics like troubleshooting and handling complex configurations (Supplementary Data S2). This iterative feedback process allowed us to adapt and prioritize future session, ensuring relevance to the evolving needs of the community. Hacky-Hours were scheduled flexibly and only held when there was clear interest, maximizing participation and avoiding unnecessary strain on communication channels.

## Rule 7: Structured and Personalized Training

To accommodate diverse skill levels and learning styles, a successful capacity-building program should combine structured group training with personalized mentoring, such as bootcamps and mentoring session, respectively. Bootcamps are an efficient way to immerse participants in the theoretical and practical aspects, exposing them to the tools and techniques essential for modern bioinformatics. While mentoring sessions are a personalized approach that empower individuals to navigate the complexities of bioinformatics.

At The Kids, we implemented an integrated approach that included immersive week-long bootcamps alongside tailored one-to-one mentorship sessions. Bootcamps effectively introduced participants, especially students and EMCRs, to the theoretical and practical aspects of Nextflow and nf-core. These events balanced conceptual learning with hands-on pipeline development and included dedicated “Bring Your Own Data” (BYO-D) sessions to ensure relevance to individual projects. Including IT team members in these sessions fostered mutual understanding between researchers and technical support, aligning infrastructure solutions with research needs.

To complement group training, we offered one-to-one mentoring tailored to participants with limited bioinformatics experience or specific project challenges. Mentors from the organizing team provided individualized support, helping participants gain confidence in using workflow tools. These sessions also generated valuable feedback, which shaped and expanded the BioWiki documentation (https://github.com/TelethonKids/Nextflow-BioWiki), ensuring it remained practical and user-informed (McGrath et al., 2019). For broader reach, we recommend starting with foundational seminars (Rule 5) and Hacky-Hours (Rule 6) before launching advanced bootcamps focused on troubleshooting and complex configurations (Hagan et al., 2020). This layered approach promotes continuous learning and maximizes long-term impact.

## Rule 8: Facilitate continuous learning and engagement

To ensure the long-term success of the capacity development program and to build a community of “power-users,” it is crucial to establish continual learning and engagement opportunities. Leveraging internal platforms, such as messaging apps or newsletters, is an effective strategy for informing participants about current activities and upcoming events. A monthly newsletter is also an effective medium for sharing updates on Nextflow developments, like module binaries and wave containers, and nf-core community projects, including new pipelines, modules, and configurations.

At The Kids, we established a Microsoft Teams channel as a space for discussion and promoted a local volunteer community. The long-term success of the program depends on users willing to assist each other in areas such as creating new software/pipelines (Brack et al., 2022), customizing existing pipelines, addressing infrastructure questions, and designing bioinformatics experiments within the infrastructure. While Microsoft Teams was effective in our local context, we acknowledge that integrated environments, such as Slack and Jira (https://support.atlassian.com/jira-product-discovery/docs/configure-the-slack-integration/) may offer more scalable solution for larger or international teams. Jira is a widely used project management platform use to track tasks and progress in software and scientific projects. It can be connected to Slack through both Jira Cloud and Jira Server, helping teams streamline communication, version control and issue tracking in one shared space.

Beyond the internal communication, engagement with the global Nextflow and nf-core communities is also encouraged and should be a major goal of the capacity-building program for long-term success. Communities such as online forums offer valuable resources, this is demonstrated in the nf-core Slack group (https://nf-co.re/join) and the organized community events, including online training sessions (https://nf-co.re/events/hackathon). By participating in these community events, researchers gain access to a wealth of knowledge and opportunities for collaboration. We anticipate engagement with open-source communities like nf-core will become the standard for modern bioinformatics. While our capacity-building did not directedly focus on open science or FAIR (findability, accessibility, interoperability, and reusability) data principles, it supports these goals by encouraging the use of transparent, community-driven tools.

While nf-core provides a strong foundation for global engagement, other platforms such as WorkflowHub (https://about.workflowhub.eu) (Gustafsson et al., 2025) and Galaxy (https://usegalaxy.org) (Abueg et al., 2024) offer tools and infrastructure for collaborative workflow development, reproducibility, and execution, which are particularly helpful when compute funding or resources are limited. The Galaxy platform, for example, is supported by globally distributed public servers, including Europe (https://usegalaxy.eu) and Australia (https://usegalaxy.org.au). These international platforms can act as springboards for expanding local initiatives into sustainable, globally connected efforts.

## Rule 9: Assess the program design and implementation regularly

In our experience, a key factor in running a successful bioinformatics capacity development program is to conduct regular assessments. Feedback from participants provided the organizing team the opportunity to evaluate engagement levels and refine the program as needed. As the program progresses, some participants may struggle to keep on track. To address this, we recommend implementing quarterly checkpoints to ensure program objectives are being met.

These assessments should focus on three fundamental questions:

1. What is working?
2. What can be improved?
3. What should be discontinued in the next phase?

At The Kids, we used the initial survey (Rule-3) as a baseline and conducted regular evaluations to gather participant feedback (Supplementary Data S2). This enabled us to track learning progression, identify challenges, and refine training initiatives. Adjustments to internal communication strategies were also made to ensure the program continued to meet the needs of EMCRs and aligned with the institute’s research goals. Our final survey provided valuable insights into the program’s effectiveness and participant engagement (Supplemental Data S3). It assessed whether we met our objectives and successfully enhanced EMCR skills. Additionally, the final survey gave participants an opportunity to reflect on their learning, reinforcing knowledge retention and paving the way for future capacity-building initiatives.

## Conclusion

In summary, our experience at The Kids demonstrates that integrating Nextflow and nf-core into bioinformatics training significantly enhances research capacity. These platforms equip EMCRs to navigate complex “omics” data analysis by providing transparent and standardized workflows. Through tailored implementation aligned with our computing environments and research needs, we have improved technical capabilities while fostering a culture of continual learning. Although many EMCRs are still analyzing data or preparing manuscripts, we have already observed a measurable impact, with several researchers incorporating nf-core pipelines or Nextflow workflows into published studies (Agudelo-Romero et al., 2024; 2025; Hancock et al., 2025; Montgomery et al., 2025; Sharma et al., 2025a; 2025b). These outputs reflect increased bioinformatics proficiency and demonstrate how the program has strengthened research capacity across disciplines.

A key factor in this sustained impact is the emphasis on reproducibility and traceability, cornerstones of reliable scientific practice. Crucially, Nextflow and nf-core not only support reproducibility, by recording parameters, software versions and environments, but also now offer native support for data provenance through features like Nextflow’s data lineage module. This feature enables results to be traced back to specific inputs, pipeline version, and configuration settings, providing transparent file-level provenance in a structured format. Separately, nf-core pipeline templates include automatic generation of MultiQC reports, which summarises pipeline-level metrics, software versions, resource usage, and parameters. While the data lineage module and MultiQC report serve different purposes, they complement each other in promoting reproducible and auditable workflows. For teams requiring more advance auditability and persistent provenance, we also highlight platforms such as Trecode (Kerstens et al., 2023) and Terra.bio (https://app.terra.bio). Together, these tools help ensure our research is not only reproducible but also transparently traceable and robust for long-term use and compliance.

Investing in awareness campaigns, strategic planning, community engagement, and varied training opportunities, including seminars, Hacky-Hours, mentorship programs, and bootcamps, yields significant benefits. By fostering continuous learning and engagement, we have upskilled researchers and built sustainable bioinformatics documentation and teaching materials. We identified several opportunities for enhancing the program and are excited to share the following recommendations for those planning similar initiatives (Supplementary Table S2). Our program serves as a practical roadmap for institutions aiming to strengthen bioinformatics capabilities and support innovative research through the use of standardized, community-driven workflows.

## Data Availability

The original contributions presented in the study are included in the article/Supplementary Material, further inquiries can be directed to the corresponding author.
