# Supplementary material for: Advancing bioinformatics capacity through Nextflow and nf-core: lessons from an early-to mid-career researchers–focused program at The Kids Research Institute Australia
Source: Front Bioinform. 2025 Aug 29;5:1610015. doi: 10.3389/fbinf.2025.1610015 (PMC12425987; doi:10.3389/fbinf.2025.1610015)
Supplement: Supplementary file 1 [file Supplementaryfile1.pdf]

frontiers

Advancing Bioinformatics Capacity through Nextflow and nf-core: Lessons from an Early- to Mid-Career Researchers–Focused Program at The Kids Research Institute Australia.

PatriciaAgudelo-Romero[1,2,3,*,#], Talya Conradie[1], Jose A. Caparros-Martin[1,4,5], David J. Martino[1], Anthony Kicic[1,6,7], Stephen M. Stick[7,8], Christopher Hakkaart[9], Abhinav Sharma[10,#], and the Theme Collaboration Group.

[1]Wal-Yan Respiratory Research Centre, The Kids Research Institute Australia, Perth, Western Australia, Australia.

[2]Australian Research Council Centre of Excellence in Plant Energy Biology, School of Molecular Sciences, The University of Western Australia, Perth, Western Australia, Australia.

[3]European Virus Bioinformatics Center, Friedrich-Schiller-Universitat Jena, Thuringia, Germany.

[4]Curtin Health Innovation Research Institute (CHIRI), Curtin University, Perth, Western Australia, Australia.

[5]School of Medicine, The University of Western Australia, Perth, Western Australia, Australia.

[6]School of Population Health, Curtin University, Perth, Western Australia, Australia

[7]Centre for Cell Therapy and Regenerative Medicine, Medical School, The University of Western Australia, Perth, Western Australia, Australia.

[8]Department of Respiratory and Sleep Medicine, Perth Children's Hospital, Perth, Western Australia, Australia.

[9]Seqera Labs, Barcelona, Catalonia, Spain.

[10]DSI-NRF Centre of Excellence for Biomedical Tuberculosis Research; SAMRC Centre for Tuberculosis Research; Division of Molecular Biology and Human Genetics, Faculty of Medicine and Health Sciences, Stellenbosch University, Cape Town, Western Cape, South Africa.

*** Correspondence:**
Corresponding Author: Patricia.AgudeloRomero@thekids.org.au

# Co-senior authorship

Supplementary Material

Supplementary Data S1 | Theme Collaboration Award members.

Supplementary Data S2 | Initial, Regular and Final Feedback Surveys for Capacity-Building Assessment.

Supplementary Data S3 | Schedule for Nextflow Basic and Advanced Bootcamps.**Supplementary Data S4 |** Non-Exhaustive List of National (Australia) and International Grant Calls for Capacity Building.

Supplemental Table S1 | Academic usage credits from cloud computing providers

Supplemental Table S2 | Recommendations for Future Programs

Supplementary Data S1 | Theme Collaboration Award Group (Theme Collaboration Group)

Alison McDonnell, Senior Researcher.

Anya Jones, Early- and Mid-career Researcher.

Carlos Aya-Bonilla, Early- and Mid-career Researcher.

Claudia Floreani, Early- and Mid-career Researcher.

Guillaume Drouart, Early- and Mid-career Researcher.

Holly Martin, Early- and Mid-career Researcher.

Janessa Pickering, Early- and Mid-career Researcher.

Jesee Armitage, Early- and Mid-career Researcher.

Jimmy Breen, Senior Researcher.

Kak-Ming Ling, Early- and Mid-career Researcher.

Mark Nicol, Senior Researcher.

Nelly Amenyogbe, Early- and Mid-career Researcher.

Omar Elaskalani, Early- and Mid-career Researcher.

Raelene Endersby, Senior Researcher.

Sam Buckberry, Senior Researcher.

Sebastien Malinge, Senior Researcher.

Timothy Barnett, Senior Researcher.

Valerie Verhasselt, Senior Researcher.

Supplementary Data S2 | Initial, Final, and Regular Feedback Surveys for Capacity-Building Assessment.

Initial Survey

* This form will record your name, please fill your name.

About you

1. First Name

2. Last Name

3. How would you describe your current position?

- ◯ Early Career Researcher (1 to 5 years postdoc)
- ◯ Mid Career Researcher (5 to 15 years postdoc)
- ◯ Student
- ◯ Research Assistant
- ◯ Other

4. Which theme do you belong to?

- ◯ Aboriginal Health
- ◯ Brain & Behaviour
- ◯ Chronic and Severe Diseases
- ◯ Early Environment
- ◯ Other

Prior Knowledge Base

5. Have you attended a training or viewed Nextflow training material before?

   - ◯ Yes
   - ◯ No

6. Have you used nf-core pipelines before?

   - ◯ Yes
   - ◯ No

7. Have you written a pipeline using Nextflow?

   - ◯ Yes
   - ◯ No

8. Have you contributed to nf-core before?

   - ◯ Yes
   - ◯ No

9. Are you a member of the nf-core Slack?

   - ◯ Yes
   - ◯ No

10. Are you a member of the Nextflow Slack?

    - ◯ Yes
    - ◯ No

11. Are you familiar with Git and Github?

    - ◯ Yes
    - ◯ No

12. Do you have access to a server or cluster for data analysis?

- Yes
- No

Core Interests

13. Which topics are you interested in?

- Converting existing pipelines to Nextflow
- Writing new pipelines in Nextflow
- Gaining clarity on Nextflow
- Using the infrastructure efficiently
- Engaging with Nextflow and nf-core communities
- Other

14. I am confident using bash

15. I am confident using git and GitHub for version control

16. I am confident writing in markdown

17. I am confident writing pipelines with Nextflow

18. I am confident running nf-core pipelines

This content is neither created nor endorsed by Microsoft. The data you submit will be sent to the form owner.

Microsoft Forms

Quarterly Feedback

* This form will record your name, please fill your name.

About you

1. First Name

2. Last Name

3. How would you describe your current position?

- ◯ Early Career Researcher (1 to 5 years postdoc)
- ◯ Mid Career Researcher (5 to 15 years postdoc)
- ◯ Student
- ◯ Research Assistant
- ◯ Other

4. Which theme do you belong to?

- ◯ Aboriginal Health
- ◯ Brain & Behaviour
- ◯ Chronic and Severe Diseases
- ◯ Early Environment
- ◯ Other

Feedback for Organizers

5. I am happy with the content covered in the seminars

☆ ☆ ☆ ☆ ☆

6. I am happy with the delivery of the content in the seminars

☆ ☆ ☆ ☆ ☆

7. I am confident in running Nextflow pipelines

☆ ☆ ☆ ☆ ☆

8. I am confident in writing pipelines with Nextflow

☆ ☆ ☆ ☆ ☆

frontiers

Feedback

9. What can be improved in the seminars?

10. Have you been using the internal documentation?

11. Have you engaged with the Nextflow or nf-core community Slack?

12. Would you be interested in Hacky-hours or One-to-one mentoring? If so, on which topics?

13. Any Other Comment

This content is neither created nor endorsed by Microsoft. The data you submit will be sent to the form owner.

Microsoft Forms

Final Survey

* This form will record your name, please fill your name.

About you

1. First Name

2. Last Name

3. How would you describe your current position?

- ◯ Early Career Researcher (1 to 5 years postdoc)
- ◯ Mid Career Researcher (5 to 15 years postdoc)
- ◯ Student
- ◯ Research Assistant
- ◯ Other

4. Which theme do you belong to?

- ◯ Aboriginal Health
- ◯ Brain & Behaviour
- ◯ Chronic and Severe Diseases
- ◯ Early Environment
- ◯ Other

Current Knowledge Base

5. The program help you adopt open and reproducible data analysis methods in your research?

◯ Yes

◯ No

6. Are you using nf-core pipelines in your routine research?

◯ Yes

◯ No

7. Are you using nf-core best practices while writing your own pipelines?

◯ Yes

◯ No

9. Are you a member of the nf-core Slack?

◯ Yes

◯ No

10. Are you a member of the Nextflow Slack?

◯ Yes

◯ No

11. Are you using Git and GitHub for collaboration and pipeline development?

◯ Yes

◯ No

Core Interests

13. In which areas you have benefited the most after participating in this program?

- ◯ Converting existing pipelines to Nextflow
- ◯ Writing new pipelines in Nextflow
- ◯ Gaining clarity on Nextflow
- ◯ Using the infrastructure efficiently
- ◯ Engaging with Nextflow and nf-core communities
- ◯ Other

Supplementary Data S3 | Schedule for Nextflow Basic and Advanced Bootcamps.

Initial Bootcamp

**Duration**	**Day-1**	**Day-2**	**Day-3**	**Day-4**	**Day-5**
1 hour	Introduction to computers and institute infrastructure	Day 1 review and questions	Day 2 review and questions	Day 3 review and questions.	Day 4 review and questions.
1 hour	Introduction to Linux and command line	Introduction to nf-core	nf-core pipeline configuration and deployment	Toy pipeline development-1	Remote monitoring and cloud execution on Seqera Platform.
1 hour	*BREAK*	*BREAK*	*BREAK*	*BREAK*	*BREAK*
1 hour	Introduction to Nextflow	Introduction to package management and containers for reproducible computing	Designing computational experiments; analyzing and storing data	Toy pipeline development-2	Bring your own data
1 hour		Bring your own data	Bring your own data	Bring your own data	

Advanced Bootcamp

**Duration**	**Day-1**	**Day-2**	**Day-3**	**Day-4**	**Day-5**
1 hour	Introduction to the institute's infrastructure and policies	Introduction to nf-core tools	Building nf-core pipelines and customization	Review of previous concepts.	Bring your own data
1 hour	Introduction to nf-core	Introduction to nf-core modules and subworkflows			

		project			
1 hour	*BREAK*	*BREAK*	*BREAK*	*BREAK*	*BREAK*
1 hour	Deploying nf-core standard pipelines (nf-core/fetchngs)	Introduction to nf-core standard pipeline template	Building nf-core pipelines and customization-2	Designing computational experiments with multiple pipelines	Bring your own data
1 hour	Question and Answer	Question and Answer	Bring your own data	Bring your own data	

frontiers

Supplementary Data S4 | Non-Exhaustive List of National (Australia) and International Grant Calls for Capacity Building

AUSTRALIA:

Government Funding:

- **Australian Research Council (ARC):**
    1. **Discovery Grants:** These grants support fundamental research projects in any field of science, including bioinformatics.
    2. **Linkage Grants:** These grants support collaborative research projects between universities and industry or other organizations.
- **National Health and Medical Research Council (NHMRC)**
- **Medical Research Future Fund (MRFF)**

Other Funding Sources:

- **Australian BioCommons**
- **Bioplatforms Australia**
- **International Funding Bodies**: Such as European Research Council, United States Agency for International Development, African Academy of Sciences, African Network of Sciences, African Bioinformatics Network. Latin American Network for Genomic Sciences, Latin American Bioinformatics Network**,** Brazilian Network for Genomic Sciences (BRAG)
- **National Funding Bodies:** National Council for Scientific and Technological Development (CNPq), Indian Council of Medical Research (ICMR), China National Natural Science Foundation (NSFC)
- **Universities and Research Institutions:** The university or the research institute might have its own programme for capacity building of its staff and visitors.
- **Industry Partners:** Industry partners may be interested in funding research projects that have the potential to benefit their businesses.

Supplemental Table S1 | Academic usage credits from cloud computing providers

Cloud Provider	Academic credits
**Google Cloud Platform (GCP)**	● **Google Cloud Research Credits**: Researchers can apply for credits to support their research projects.<br>● **Google Cloud for Education**: This program offers free credits, training, and resources for students and educators.
**Amazon Web Services (AWS)**	● **AWS Educate**: This program provides free credits, training, and resources for students and educators.<br>● **AWS Research Credits**: Researchers can apply for credits to support their research projects.
**IBM Cloud**	● **IBM Cloud Academic Initiative**: This program provides free credits, training, and resources for students and educators.<br>● **IBM Research Cloud**: This program provides free credits and resources for researchers.
**Oracle Cloud Infrastructure (OCI)**	● **Oracle Cloud Infrastructure for Education**: This program offers free credits, training, and resources for students and educators.
**Microsoft Azure**	● **Azure for Research**: This program provides free credits, training, and resources for researchers.<br>● **Microsoft Imagine**: This program offers free credits, training, and resources for students and educators.

Supplemental Table S2 | Recommendations for Future Programs

**Recommendation**	**Description**
**Secure Adequate Funding**	We recommend a cross-disciplinary approach to secure funding and align with institutional goals (Supplemental Data S4). Funding determines the scope and depth of the program. At The Kids, an internal Theme Collaboration Award supported the identification of bioinformatics needs across three disciplines (Chronic and Severe Diseases, Early Environment, and Indigenous Health), involving 12 research groups across two sites (Perth and Adelaide) (Supplemental Data S1).
**Structure Bootcamps Strategically**	Organize two bootcamps: a foundational one to introduce bioinformatics basics and an advanced one for developing and customizing pipelines (Supplemental Data S3). This ensures participants build core skills before tackling more complex tasks independently.
**Engage with national infrastructures.**	Partner with national infrastructures to ensure the long-term sustainability of the best practices. At The Kids, we benefited from partnerships with the Australian Pawsey Supercomputing Research Centre (Pawsey Supercomputing Research Centre Perth, 2023a, 2023b, 2023c, 2023d,) and the Australian BioCommons Leadership Share (ABLES) program (Gustafsson et al., 2023) which provided access to cutting-edge high-performance computing resources, including long-term storage.
**Facilitate Round-Table Discussions**	Host monthly expert-led round-tables to share best practices, explore case studies, offer troubleshooting advice, and reinforce the value of the bioinformatics community.
